# Precise Measurement of Lunar Spectral Irradiance at Visible Wavelengths

**DOI:** 10.6028/jres.118.020

**Published:** 2013-10-29

**Authors:** CE Cramer, KR Lykke, JT Woodward, AW Smith

**Affiliations:** 1 National Institute of Standards and Technology, Gaithersburg, MD 20899; 2 L-1 Standards and Technology, Inc., New Windsor, MD 21776

**Keywords:** calibration, lunar spectral irradiance, moon, radiometry, remote sensing

## Abstract

We report a measurement of lunar spectral irradiance with an uncertainty below 1 % from 420 nm to 1000 nm. This measurement uncertainty meets the stability requirement for many climate data records derived from satellite images, including those for vegetation, aerosols, and snow and ice albedo. It therefore opens the possibility of using the Moon as a calibration standard to bridge gaps in satellite coverage and validate atmospheric retrieval algorithms. Our measurement technique also yields detailed information about the atmosphere at the measurement site, suggesting that lunar observations are a possible solution for aerosol monitoring during the polar winter and can provide nighttime measurements to complement aerosol data collected with sun photometers. Our measurement, made with a novel apparatus, is an order of magnitude more accurate than the previous state-of-the-art and has continuous spectral coverage, removing the need to interpolate between filter passbands.

## 1. Introduction

Although the Moon is our nearest celestial neighbor, our knowledge of its spectral irradiance lags behind more precise measurements of the Sun and bright stars such as Vega. The most careful radiometric study of the Moon to date, made with the United States Geological Survey’s (USGS) Robotic Lunar Observatory from 1996 to 2003, is thought to have an uncertainty between 5 % and 10 % in its spectral irradiance scale [1]. Motivated by the possibility of using a better-calibrated Moon to maintain consistency in climate data records across gaps in satellite coverage, we have developed a novel apparatus to measure lunar spectral irradiance at visible wavelengths. Here, we report a measurement of lunar spectral irradiance that is traceable to Système Internationale (SI) radiometric units with a combined standard uncertainty of 1 % or less from 420 nm to 1000 nm in regions of the spectrum unaffected by strong molecular absorption.

The recent loss of the European Space Agency’s Envisat and impending seventeen-month delay between the end of the United States polar-orbiting Suomi mission and its replacement highlight the need to maintain climate data records across gaps in satellite coverage. Images from these satellites contribute to a diverse array of data records, including vegetation, snow and ice albedo, clouds, and aerosols. As climate variability and change are evident only when the time series of measurements is sufficiently long, it is essential to retain the ability to compare data on either side of a gap. The uncertainty in absolute radiometric calibration of many satellite imagers at visible wavelengths is inadequate for this purpose, placing climate data records at risk.

Current satellite missions use a combination of solar diffusers and well-characterized sites on the Earth’s surface to establish an absolute scale for measurements made in orbit. Both techniques have significant limitations. Degradation of the solar diffusers must be monitored and separated from degradation in the sensors they are meant to calibrate, limiting the combined uncertainty to > 2 % and adding an additional failure point to the system [2]. Earth surface sites typically require constant maintenance and monitoring and are therefore themselves susceptible to gaps in coverage. While stable sites such as the Libyan desert have been left unmonitored after an initial calibration, the consistency in data products dependent on these sites is at risk if there is even a small change in surface reflectance. Further, an atmospheric correction must be applied to images of the Earth’s surface, potentially conflating errors in atmospheric modeling with instrument calibration. Lunar calibration sidesteps these issues: the Moon is of similar brightness to the Earth so there is no need for a diffuser, it is above the atmosphere so a correction is not required, and the Moon is available to all satellites orbiting the Earth throughout their mission lifetimes.

Lunar calibration has already been used to track changes in sensor response as small as 0.1 %, allowing the SeaWiFS ocean color instrument to meet its stability requirement and demonstrating that it is possible to surmount the data analysis challenges in using the Moon for radiometric calibration of satellite images [3,4]. However, lunar calibration has not been a viable means of absolute calibration on orbit because uncertainty in the absolute scale of the Moon’s spectral irradiance is too large. Typical stability requirements for climate data products range from 0.1 % for ocean color and 0.8 % for vegetation to 1.5 % for aerosols [2,5,6]. The corresponding accuracy requirements – 0.5 % for ocean color, 2 % for vegetation, and 3 % for aerosols – are less exacting, but still well below the previous uncertainties on lunar irradiance.

We have developed a prototype apparatus consisting of a telescope coupled to a non-imaging spectrograph and installed it at the Fred Lawrence Whipple Observatory in southern Arizona (31 41 1.5 N, 110 52 40.8 W, 2367 m). In contrast to previous measurements, which were made with an imaging system in a set of ~10 nm-wide filter passbands, our technique provides continuous spectral coverage. The measurement we present here is derived from a time series of 233 lunar spectra collected on the night of 30 November, 2012. The spectra have one-minute spacing and span an airmass range of 1 to 3, with the Sun-Moon-Observer angle (i.e., lunar phase) ranging from 17° to 20°.

## 2. Instrumentation and Calibration

We observe the Moon with a 106 mm refracting telescope with a 50.8 mm-diameter integrating sphere placed at its focal plane, where the imaged lunar disc is approximately one-third the size of the sphere’s 12 mm entrance aperture. The sphere scrambles light from the entire lunar disc, uniformly illuminating a flexible light guide that directs the moonlight into a spectrograph with 3 nm resolution from 380 nm to 1040 nm. Spectra are recorded at one-minute intervals throughout the night with a 45-second exposure time. The time stamp on each spectrum is tied to a GPS receiver and is accurate to within 200 *μ*s.

We flux-calibrate the lunar spectra by observing a light source with known spectral irradiance that acts as an “artificial moon”. The artificial moon is a 300 mm-diameter integrating sphere placed 36.423 m from the telescope’s effective aperture and illuminated with quartz-tungsten-halogen (QTH) lamps. Atmospheric extinction is negligible over this horizontal path outside the molecular absorption bands that we do not consider in this work. The distance and lamp current are chosen to give the imaged sphere aperture a smaller size and similar brightness to the full Moon when viewed with the telescope. Repeating the calibration every two hours throughout the night, we find that the telescope calibration is stable to better than 0.2 % from 420 nm to 1000 nm.

Traceability to the SI is established by measuring the spectral irradiance of the artificial moon with a NIST-calibrated spectrograph during every telescope calibration. The calibrated spectrograph’s spectral irradiance responsivity is tied to the SI through a QTH lamp of known output [7]. The NIST QTH lamp calibration is performed before and after each deployment to the Mt. Hopkins site. The calibrations at NIST are reproducible at a level below the uncertainty in the QTH lamp calibration, which is the single largest source of uncertainty in our measurement.

## 3. Analysis

We derive the lunar spectral irradiance at the top of the atmosphere (TOA) from the time series of ground-based observations by means of the Beer-Lambert-Bougher law, which states that the observed spectral irradiance, *I*(*λ*, *t*), is equal to the TOA spectral irradiance, *I*_0_(*λ*, *t*), multiplied by the transmission through each significant component of the atmosphere:

(1) $$I(\lambda ,t)e=I_{0}(\lambda ,t)e^{-\Sigma m_{i}\tau_{i}}$$

The transmission, $e^{-m_{i}\tau_{i}}$, is defined by two parameters: a geometrical airmass function, *m_i_*, that depends on the vertical profile of the atmospheric component and lunar zenith angle, and the corresponding optical depth, *τ_i_*. Geometrical effects, including the continually-evolving relative positions of the Sun, Moon, and observer, produce the time-dependence in TOA lunar irradiance. At our site in southern Arizona, we find that it is necessary to make explicit corrections for ozone and stratospheric aerosols in addition to accounting for the ~2 % change in the TOA lunar irradiance throughout the night.

We use the model developed by the USGS [1] to account for the time dependence in TOA lunar irradiance, data from NASA’s Ozone Monitoring Instrument (OMI) [8] to correct for ozone absorption, a background profile based on nine years of data (1997–2005) from the second-generation Stratospheric Aerosol and Gas Experiment (SAGE II) [9] to correct for stratospheric aerosols, and calculate the expected Rayleigh scattering from the mean of radiosonde profiles taken within 10 days of our measurement in Tucson, the nearest site in the Integrated Global Radiosonde Archive [10]. All atmospheric calculations were performed with MODTRAN 5 [11], and solar system ephemeris calculations were performed with the NASA Jet Propulsion Laboratory’s Horizons [12].

After correcting the calibrated lunar spectra for stratospheric aerosols and ozone and accounting for the change in the TOA lunar irradiance throughout the night, we perform a linear least-squares fit on the logarithm of the spectral irradiance (left-hand side of Eq. 1) as a function of the airmass for Rayleigh scattering, *m_R_*. The intercept yields the absolute spectral irradiance at each wavelength, and the slope of the line is the Rayleigh optical depth, with the fit uncertainty accounting for statistical uncertainty in the data. Structure in the fit residuals could indicate temporal variability in the atmosphere or the presence of uncorrected atmospheric constituents. This analysis is valid in regions of the spectrum free from strong molecular absorption.

## 4. Results and Discussion

**Fig. 1:**
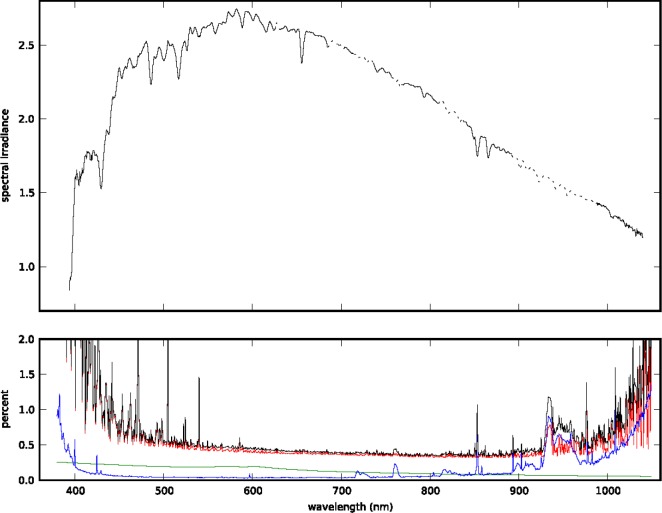
Spectral irradiance of the Moon in units of *μ*W m^−2^ nm^−1^ at 11:40:43 on 30 November, 2012 UT (top panel). The associated uncertainty in the linear fit (blue), combined uncertainty in the corrections for ozone and stratospheric aerosols (green), uncertainty in the calibration (red), and total combined uncertainty (black) are shown in the lower panel. Our measurement is valid with the uncertainty shown here in the regions of the spectrum not affected by strong molecular absorption. At wavelengths where the discrepancy between our measured Rayleigh transmission and the expected Rayleigh transmission is greater than 1 % (see Fig. 2), we scale the USGS model prediction to produce the dotted line in the upper panel.

**Fig. 2:**
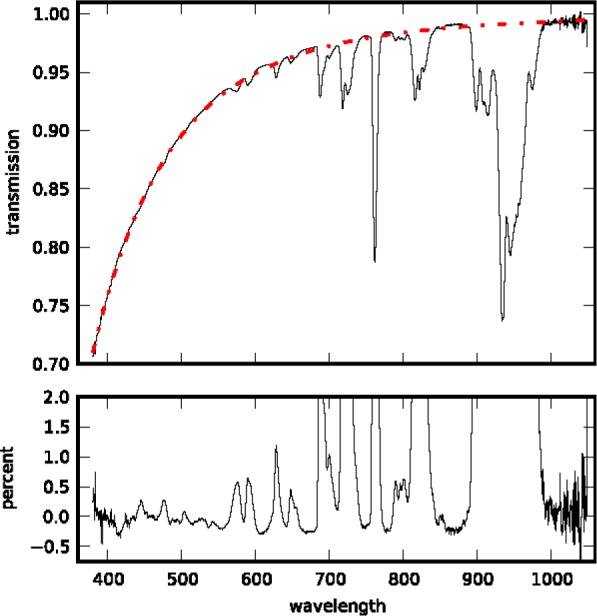
The top panel shows atmospheric transmission resulting from the Beer-Lambert-Bougher law analysis of our lunar measurements (black line) together with the Rayleigh transmission calculated from radiosonde profiles (red dash-dotted line). The lower panel shows the percent difference between measurement and calculation. The discrepancies are due to oxygen and water vapor absorption.

At wavelengths unaffected by molecular absorption, the Beer-Lambert-Bougher law fit has normally-distributed residuals and *R*^2^ > 0.998 from 400 nm to 600 nm, decreasing to 0.992 at 680 nm. This technique only yields reliable results if the atmosphere is stable and isotropic. The night of 30 November, 2012 was near-ideal: the few visible clouds in the sky dissipated at sunset, we do not see structure in the fit residuals that would indicate a thin cirrus layer, and the tropospheric aerosol optical depth was extremely low. Figure 1 shows the measured TOA irradiance at 11:40:43 on 30 November, 2012, Universal Time (UT), and Table 1 gives values for selected wavelengths. We find excellent agreement between the result of our fit and calculated Rayleigh transmission (Fig. 2). The difference between the two corresponds to a tropospheric aerosol optical depth *τ_t.a._* < 0.005. The combined standard uncertainty in the measured TOA irradiance, shown in the lower panel of Fig. 1, is less than 1 % between 420 nm and 1000 nm.

Uncertainty in the laboratory calibration of the spectrograph that establishes SI-traceability dominates the uncertainty budget except at the red and blue edges of the spectrum, where the signal-to-noise ratio in the telescope calibration is low. The second-largest term in the uncertainty budget is from the stratospheric aerosol correction. Lacking reliable stratospheric aerosol data from 2012, we have estimated a background level from earlier measurements and assumed a 25 % uncertainty in the optical depth to account for the uncertainty in those measurements and typical variation in stratospheric aerosol extinction [13]. The uncertainty in the ozone correction and linear fit, also included in Fig. 1, are small in comparison. We neglect factors such as timing uncertainty, the finite spectrograph resolution, background light, stray light (which is corrected in each spectrograph), wavelength calibration uncertainty, solar irradiance variations, and telescope tracking irregularities that contribute less than 0.1 % to the combined uncertainty.

In principle, it is possible to use this measurement to set the absolute scale of the USGS model describing the time/geometry-dependence of lunar spectral irradiance with an uncertainty that meets the radiometric calibration goals for many climate data products. In practice, the measurement we present here should be the crucial first step of a long-term program. Future measurements for different lunar phase and libration angles will better define the absolute scale of the USGS model as well as expose possible unknown systematic effects. Ideally, measurements would be made over several years to sample a wide range of phase and libration angles, as described by Kieffer and Stone [1].

Future measurements could also benefit from straightforward improvements in laboratory calibration and a higher-altitude observatory. By improving the signal-to-noise ratio at the edges of the calibration spectrum, using a laser-based technique rather than the QTH lamp to establish SI-traceability [14], and moving to a higher-altitude site where the atmosphere is carefully monitored, such as the National Oceanic and Atmospheric Administration’s Mauna Loa Observatory, lunar calibrations performed with the technique presented here may meet even the 0.1 % uncertainty requirement for ocean color across the visible spectrum.

While many satellite imaging programs avoid the regions of the spectrum affected by molecular absorption for the same reasons we do here, it is desirable to fill those gaps in the spectrum. Future measurements could avoid the need to correct for water vapor, which is highly variable and anisotropically distributed, by making measurements from a high-altitude balloon or airplane. The atmospheric transmission from such a platform, which would fly at an altitude greater than 19 km, is > 99 % over most of the visible spectrum. Operating in an environment free from water vapor absorption will facilitate extending the lunar spectral irradiance calibration further into the infrared, ensuring continuity in the data records for cloud properties, snow cover, and other climate variables dependent on short-wave infrared radiometry.

Finally, we note that satellite calibration is not the only application of an improved calibration of lunar spectral irradiance. Atmospheric aerosols are constantly monitored across the globe with a network of sun photometers, providing a set of measurements that directly address the greatest uncertainty in our understanding of the Earth’s radiative energy balance [15]. Sun photometry is limited to daytime measurements, leaving open questions about diurnal variations in aerosol levels and aerosol loading during polar winters. Berkoff et al. [16] raise the possibility of lunar photometry to monitor atmospheric aerosols when the sun is not available. An improved calibration of lunar spectral irradiance aids this effort, and the measurement technique we present is capable of providing detailed spectral information about atmospheric aerosols as the lunar cycle permits.

## 5. Conclusion

We have made an SI-traceable measurement of the Moon’s spectral irradiance with uncertainties that are an order of magnitude smaller than the previous state-of-the-art, and consistent with the ROLO model to within the uncertainties indicated in Kieffer and Stone [1]. When measurements such as the one we report here are combined with the USGS model describing the time-dependence of lunar spectral irradiance, it will be possible to assign an accurate common scale to any satellite data set containing lunar views, past or present, across gaps in satellite coverage. While lunar calibration does not solve all the problems associated with discontinuities in climate data records, it is a significant part of the solution we need in the current environment of aging spacecraft, scheduling delays, and generally tight budgets. An accurate calibration of lunar irradiance will also allow validation of atmospheric retrieval algorithms for Earth-observing satellites through a comparison of lunar images to images of calibrated Earth surface sites. Finally, the Beer-Lambert-Bougher analysis we present here is sensitive to low aerosol optical depths and capable of providing detailed spectral information about aerosol optical properties. It is therefore a promising new technique for monitoring aerosols at night and during polar winters.

## Figures and Tables

**Table 1:** Spectral irradiance of the Moon at 11:40:43 on 30 November, 2012 UT

Wavelength (nm)	Spectral irradiance (*μ*W m^−2^ nm^−1^)	Uncertainty (percent)
449.7	2.348	0.85
499.9	2.395	0.56
550.0	2.633	0.45
600.2	2.669	0.44
650.1	2.598	0.40
702.8	2.474	0.38
750.0	2.314	0.37
850.2	1.870	0.36
1000.2	1.387	0.54
